# Supplementary material for: Variation of Cancer Incidence between and within GRELL Countries
Source: Int J Environ Res Public Health. 2021 Sep 2;18(17):9262. doi: 10.3390/ijerph18179262 (PMC8431723; doi:10.3390/ijerph18179262)
Supplement: Supplementary file 1 [file ijerph-18-09262-s001.zip › ijerph-1320124-supplementary.pdf]

Table S1. Average annual person years by registry (years of registration)

Geographic entity	Male	Female	Male and Female
**Argentina**	**2624956**	**2741413**	**5366369**
Chaco (2008-2012)	534952	536277	1071229
Córdoba (2008-2012)	656892	713428	1370320
Entre Rios Province (2008-2011)	520039	540961	1061000
Mendoza (2008-2012)	845838	886322	1732160
Tierra del Fuego (2008-2012)	67235	64426	131661
**Belgium**	5317843	5531570	10849413
**Brazil**	**2077949**	**2280894**	**4358842**
Aracaju (2008-2012)	262157	301690	563847
Goiânia (2008-2012)	618519	681738	1300257
Curitiba (2008-2011)	859395	939543	1798938
Jau (2008-2012)	65460	67829	133289
Pocos de Caldas (2008-2011)	73922	78006	151928
Florianopolis (2008-2010) **	198496	212087	410583
**Chile**	**1168684**	**1170606**	**2339290**
Bío Bío Province (2008-2012)	191695	192464	384159
Concepcion (2008-2010)	491178	517176	1008354
Region of Antofagasta (2008-2010)	296507	270864	567371
Valdivia (2008-2012)	189304	190103	379407
**Colombia**	**1972039**	**2148206**	**4120245**
Cali (2008-2012) **	1073515	1171180	2244695
Bucaramanga (2008-2012)	515950	559487	1075437
Manizales (2008-2012)	184764	203682	388446
Pasto (2008-2012)	197810	213857	411667
**Costa Rica**	2300043	2234845	4534888
**Ecuador**	**2986482**	**3103292**	**6089774**
Quito (2008-2012)	784486	836775	1621261
Cuenca (2008-2012)	239497	266088	505585
Guayaquil (2008-2012)	1162325	1208654	2370979
Loja (2008-2010)	98880	105583	204463
Manabi (2008-2012)	701294	686192	1387486
**France**	**5874440**	**6253058**	**12127497**

Bas-Rhin (2008-2011)	538538	566247	1104785
Calvados (2008-2012)	330000	356737	686738
Doubs (2008-2012)	260538	269010	529547
Gironde (2008-2012)	698333	758225	1456558
Haut-Rhin (2008-2012)	369596	382833	752429
Hérault (2008-2012)	498892	548010	1046902
Isère (2008-2012)	600719	619349	1220068
Lille-Métropole (2008-2012)	382610	417332	799942
Limousin (2009-2012)	181384	197175	378559
Loire-Atlantique (2008-2012)	628633	666055	1294688
Manche (2008-2011)	243410	256207	499617
Somme (2008-2012)	277891	294583	572473
Tarn (2008-2012)	183786	196718	380504
Territoire de Belfort (2008-2012)	71726	71745	143471
Vendée (2008-2012)	312729	325004	637734
Martinique	181924	211844	393768
French Guiana (2008-2012)	113732	115982	229715
**Italy**			
Ferrara (2008-2011)	171696	187460	359156
Friuli-Venezia-Giulia (2008-2010)	599559	637071	1236630
Modena (2008-2012)	330353	348295	678648
Parma (2008-2012)	205418	220110	425527
Piacenza (2008-2011)	139502	147038	286540
Reggio Emilia (2008-2012)	260367	268642	529010
Romagna (2008-2012)	597601	630355	1227956
Sud Tirolo (2008-2010)	248087	254495	502582
Trento (2008-2010)	251034	263077	514111
Veneto (2008-2010)	1244623	1319312	2563935
Val d'Aosta (2008-2012)	62350	64802	127152
Bergamo (2008-2012)	536216	545388	1081604
Biella (2008-2012)	88224	96391	184615
Como (2008-2011)	285608	298756	584364
Cremona (2008-2010)	177218	184746	361963
Lecco (2008-2010)	163912	169941	333853
Lombardy, South Pavia (2008-2010)	264804	280480	545284

Mantua (2008-2010)	196124	206611	402735
Milan (2008-2012)	1319796	1432957	2752753
Monza (2008-2012)	410086	427279	837365
Sondrio (2008-2012)	89076	92937	182013
Turin (2008-2012)	1080631	1163274	2243905
Varese (2008-2012)	418953	444620	863573
Florence & Prato (2008-2010)	577579	626388	1203967
Latina (2008-2012)	269284	280537	549821
Umbria (2008-2011)	421366	455031	876398
Barletta (2008-2011)	192619	197568	390187
Caserta (2008-2010)	440252	463756	904008
Naples (2008-2012)	276019	289231	565251
Taranto (2008-2011)	283597	301289	584886
Catania, Messina & Enna (2008-2012)	920365	990501	1910866
Nuoro (2008-2012)	107013	110718	217731
Palermo (2008-2012)	599461	645848	1245310
Ragusa & Caltanissetta (2008-2012)	282736	298948	581684
Sassari (2008-2011)	239950	249266	489216
Syracuse (2008-2012)	195913	203660	399573
**Peru, Lima**	**4536512**	**4768407**	**9304919**
**Portugal, Azores**	**121690**	**125191**	**24688**
**Spain**	**6179374**	**6252041**	**12431416**
Asturias (2008-2010)	505659	552129	1057788
Basque Country (2008-2012)	1058904	1110937	2169841
Castellón (2008-2012)	302512	299567	602080
Girona (2008-2012)	375771	366750	742521
La Rioja (2008-2012)	160369	159379	319747
Mallorca (2008-2011)	430766	428350	859116
Navarra (2008-2010)	314730	314563	629293
Tarragona (2008-2012)	404773	394496	799269
Albacete (2008-2010)	199863	198437	398300
Canary Isles (2008-2011)	866740	878524	1745265
Ciudad Real (2008-2011)	261574	262955	524528
Cuenca (2008-2011)	109505	106444	215949
Granada (2008-2012)	454037	461496	915532

Murcia (2008-2010)	734171	718016	1452187
**Switzerland**	**1100768**	**1156227**	**2256995**
Fribourg (2008-2012)	137559	138867	276426
Geneva (2008-2012)	218482	235165	453647
Neuchâtel (2008-2012)	83566	88409	171974
Ticino (2008-2012)	162546	173615	336161
Valais (2008-2012)	154422	157992	312414
Vaud (2008-2012)	344193	362179	706372
**Uruguay**	**1640405**	**1754983**	**3395387**
**Puerto Rico**	**1823075**	**1981028**	**3804103**

Table S2. Overall, highest and lowest incidence (ASRw) in males, with r/R, for each geographic entity

**Geographic entity ***	**ASRw**			r/R (%)
	Overall	Highest	Lowest	
**Argentina**	**215.22**	**237.96**	**173.24**	**30.07**
North	*189.68*	N/A	N/A	N/A
Chaco (2008-2012)	189.68	N/A	N/A	N/A
Central	*222.12*	*237.96*	*213.80*	*10.88*
Córdoba (2008-2012)	223.13	N/A	N/A	N/A
Entre Rios Province (2008-2011)	237.96	N/A	N/A	N/A
Mendoza (2008-2012)	213.8	N/A	N/A	N/A
South	*173.24*	N/A	N/A	N/A
Tierra del Fuego (2008-2012)	173.24	N/A	N/A	N/A
**Belgium**	**364.28**	N/A	N/A	N/A
**Brazil**	**272.58**	**380.33**	**230.10**	**55.11**
North-east	281.88	N/A	N/A	N/A
Aracaju (2008-2012)	281.88	N/A	N/A	N/A
Central	*301.67*	N/A	N/A	N/A
Goiânia (2008-2012)	301.67	N/A	N/A	N/A
South	*254.16*	*380.33*	*2301.0*	*39.50*
South without Florianopolis	*235.58*	*261.48*	*230.10*	*13.30*
Curitiba (2008-2011)	230.10	N/A	N/A	N/A
Jau (2008-2012)	261.48	N/A	N/A	N/A
Pocos de Caldas (2008-2011)	259.19	N/A	N/A	N/A

Florianopolis (2008-2010) **	*380.33*	N/A	N/A	N/A
**Chile**	**209.75**	**221.59**	**208.14**	**6.41**
Bío Bío Province (2008-2012)	209.27	N/A	N/A	N/A
Concepcion (2008-2010)	208.14	N/A	N/A	N/A
Region of Antofagasta (2008-2010)	221.59	N/A	N/A	N/A
Valdivia (2008-2012)	208.49	N/A	N/A	N/A
**Colombia**	**179.15**	**204.49**	**133.54**	**39.60**
Cali (2008-2012) **	*204.49*	N/A	N/A	N/A
Rest of Columbia	*149.97*	*155.94*	*133.54*	*14.94*
Bucaramanga (2008-2012)	153.51	N/A	N/A	N/A
Manizales (2008-2012)	155.94	N/A	N/A	N/A
Pasto (2008-2012)	133.54	N/A	N/A	N/A
**Costa Rica**	**173.9**	N/A	N/A	N/A
**Ecuador**	**135.11**	**192.79**	**89.45**	**76.49**
North-Central	*192.79*	N/A	N/A	N/A
Quito (2008-2012)	192.79	N/A	N/A	N/A
Central	*125.43*	*152.68*	*120.06*	*26.01*
Cuenca (2008-2012)	152.68	N/A	N/A	N/A
Guayaquil (2008-2012)	120.06	N/A	N/A	N/A
South	*168.50*	N/A	N/A	N/A
Loja (2008-2010)	168.50	N/A	N/A	N/A
Pacific				
Manabi (2008-2012)	89.45	N/A	N/A	N/A
**France**	**369.35**	**432.04**	**303.96**	**34.68**
Metropolitan	*372.25*	*432.04*	*334.45*	*26.20*
Bas-Rhin (2008-2011)	370.2	N/A	N/A	N/A
Calvados (2008-2012)	376.97	N/A	N/A	N/A
Doubs (2008-2012)	388.8	N/A	N/A	N/A
Gironde (2008-2012)	365.38	N/A	N/A	N/A
Haut-Rhin (2008-2012)	366.53	N/A	N/A	N/A
Hérault (2008-2012)	357.34	N/A	N/A	N/A
Isère (2008-2012)	362.66	N/A	N/A	N/A
Lille-Métropole (2008-2012)	432.04	N/A	N/A	N/A
Limousin (2009-2012)	364.54	N/A	N/A	N/A
Loire-Atlantique (2008-2012)	396.93	N/A	N/A	N/A

Manche (2008-2011)	355.74	N/A	N/A	N/A
Somme (2008-2012)	368.77	N/A	N/A	N/A
Tarn (2008-2012)	334.45	N/A	N/A	N/A
Territoire de Belfort (2008-2012)	385.81	N/A	N/A	N/A
Vendée (2008-2012)	365.04	N/A	N/A	N/A
Overseas	*304.79*	*312.08*	*303.96*	*2.66*
Martinique	303.96	N/A	N/A	N/A
French Guiana (2008-2012)	312.08	N/A	N/A	N/A
**Italy**	**331.81**	**388.53**	**271.96**	**35.13**
North-east	*346.53*	*372.44*	*297.06*	*21.75*
Ferrara (2008-2011)	372.44	N/A	N/A	N/A
Friuli-Venezia-Giulia (2008-2010)	354.6	N/A	N/A	N/A
Modena (2008-2012)	347.81	N/A	N/A	N/A
Parma (2008-2012)	348.95	N/A	N/A	N/A
Piacenza (2008-2011)	339.77	N/A	N/A	N/A
Reggio Emilia (2008-2012)	319.51	N/A	N/A	N/A
Romagna (2008-2012)	358.38	N/A	N/A	N/A
Sud Tirolo (2008-2010)	333.54	N/A	N/A	N/A
Trento (2008-2010)	297.06	N/A	N/A	N/A
Veneto (2008-2010)	346.76	N/A	N/A	N/A
North-west	*343.67*	*388.53*	*330.74*	*16.82*
Val d'Aosta (2008-2012)	343.87	N/A	N/A	N/A
Bergamo (2008-2012)	365.48	N/A	N/A	N/A
Biella (2008-2012)	388.53	N/A	N/A	N/A
Como (2008-2011)	336.12	N/A	N/A	N/A
Cremona (2008-2010)	349.03	N/A	N/A	N/A
Lecco (2008-2010)	348.18	N/A	N/A	N/A
Lombardy, South Pavia (2008-2010)	353.85	N/A	N/A	N/A
Mantua (2008-2010)	346.11	N/A	N/A	N/A
Milan (2008-2012)	346.53	N/A	N/A	N/A
Monza (2008-2012)	330.74	N/A	N/A	N/A
Sondrio (2008-2012)	379.92	N/A	N/A	N/A
Turin (2008-2012)	331.2	N/A	N/A	N/A
Varese (2008-2012)	336.17	N/A	N/A	N/A
Central	*324.55*	*337.07*	*300.37*	*11.31*

Florence & Prato (2008-2010)	337.07	N/A	N/A	N/A
Latina (2008-2012)	300.37	N/A	N/A	N/A
Umbria (2008-2011)	328.67	N/A	N/A	N/A
South	*317.03*	*339.23*	*295.26*	*13.87*
Barletta (2008-2011)	295.26	N/A	N/A	N/A
Caserta (2008-2010)	324.86	N/A	N/A	N/A
Naples (2008-2012)	339.23	N/A	N/A	N/A
Taranto (2008-2011)	300.8	N/A	N/A	N/A
Islands	*292.27*	*311.26*	*271.96*	*13.45*
Catania, Messina & Enna (2008-2012)	295.29	N/A	N/A	N/A
Nuoro (2008-2012)	311.26	N/A	N/A	N/A
Palermo (2008-2012)	298.24	N/A	N/A	N/A
Ragusa & Caltanissetta (2008-2012)	271.96	N/A	N/A	N/A
Sassari (2008-2011)	298.1	N/A	N/A	N/A
Syracuse (2008-2012)	272.49	N/A	N/A	N/A
**Peru, Lima**	**187.04**	N/A	N/A	N/A
**Portugal, Azores**	**381.92**	N/A	N/A	N/A
**Spain**	**339.97**	**382.91**	**279.90**	**30.30**
North-East	*357.91*	*382.91*	*317.21*	*18.36*
Asturias (2008-2010)	361.27	N/A	N/A	N/A
Basque Country (2008-2012)	382.91	N/A	N/A	N/A
Castellón (2008-2012)	317.21	N/A	N/A	N/A
Girona (2008-2012)	335.79	N/A	N/A	N/A
La Rioja (2008-2012)	337.72	N/A	N/A	N/A
Mallorca (2008-2011)	347.92	N/A	N/A	N/A
Navarra (2008-2010)	360.94	N/A	N/A	N/A
Tarragona (2008-2012)	346.39	N/A	N/A	N/A
South	308.34	337.66	279.9	18.73
Albacete (2008-2010)	285.48	N/A	N/A	N/A
Canary Isles (2008-2011)	306.09	N/A	N/A	N/A
Ciudad Real (2008-2011)	296.01	N/A	N/A	N/A
Cuenca (2008-2011)	279.9	N/A	N/A	N/A
Granada (2008-2012)	303.66	N/A	N/A	N/A
Murcia (2008-2010)	337.66	N/A	N/A	*Na*
**Switzerland**	**338.22**	**350.38**	**310.39**	**11.82**

Fribourg (2008-2012)	346.51	N/A	N/A	*Na*
Geneva (2008-2012)	344.76	N/A	N/A	N/A
Neuchâtel (2008-2012)	328.28	N/A	N/A	N/A
Ticino (2008-2012)	310.39	N/A	N/A	N/A
Valais (2008-2012)	350.38	N/A	N/A	N/A
Vaud (2008-2012)	343.32	N/A	N/A	N/A
**Uruguay**	**289.56**	**286.34**	**292.82**	N/A
**Puerto Rico**	**258.28**	N/A	N/A	N/A

*Countries in bold, regions in italics, cancer registries in normal font. Regions may be a group of geographically related cancer registries or a single registry (if responsible for wide within region incidence variation).

** Registry responsible for /R ≥30 in country/region

N/A: geographic entity not split into sub-entities because r/R <30.

Table S3. Overall, highest and lowest incidence (ASRw) in females with r/R, for each geographic entity

**Geographic entity**	**ASRw**			r/R(%)
	Overall	Highest	Lowest	
**Argentina**	**201.16**	**213.54**	**149.16**	**32.00**
North	*174.77*	N/A	N/A	N/A
Chaco (2008-2012)	174.77	N/A	N/A	N/A
Central	*207.66*	*213.54*	*197.74*	*7.61*
Córdoba (2008-2012)	213.54	N/A	N/A	N/A
Entre Rios Province (2008-2011)	197.74	N/A	N/A	N/A
Mendoza (2008-2012)	207.93	N/A	N/A	N/A
South	*149.16*	N/A	N/A	N/A
Tierra del Fuego (2008-2012)	149.16	N/A	N/A	N/A
**Belgium**	**289.21**	N/A	N/A	N/A
**Brazil**	**199.34**	**338.43**	**172.68**	**83.15**
North-east	208.57	*208.57*	*174.76*	*16.80*
Aracaju (2008-2012)	208.57	N/A	N/A	N/A
Central	*194.18*	N/A	N/A	N/A
Goiânia (2008-2012)	194.18	N/A	N/A	N/A
South	*200.10*	338.43	172.68	*82.83*
South without Florianopolis	*179.99*	*181.19*	*172.68*	*4.72*
Curitiba (2008-2011)	181.19	N/A	N/A	N/A
Jau (2008-2012)	174.76	N/A	N/A	N/A
Pocos de Caldas (2008-2011)	172.68	N/A	N/A	N/A
Florianopolis (2008-2010) **	338.43	N/A	N/A	N/A
**Chile**	**174.99**	**178.59**	**168.36**	**5.85**
Bío Bío Province (2008-2012)	177.92	N/A	N/A	N/A
Concepcion (2008-2010)	178.59	N/A	N/A	N/A
Region of Antofagasta (2008-2010)	173.73	N/A	N/A	N/A
Valdivia (2008-2012)	168.36	N/A	N/A	N/A
**Colombia**	**171.71**	**164.84**	**144.36**	**11.93**
Cali (2008-2012)	185.4	N/A	N/A	N/A
Bucaramanga (2008-2012)	156.19	N/A	N/A	N/A
Manizales (2008-2012)	164.84	N/A	N/A	N/A
Pasto (2008-2012)	144.36	N/A	N/A	N/A
**Costa Rica**	**167.25**	N/A	N/A	N/A

**Ecuador**	**152.43**	**206.51**	**102.32**	**68.35**
North-Central	*199.11*	N/A	N/A	N/A
Quito (2008-2012)	199.11	N/A	N/A	N/A
Central	*144.88*	*149.44*	*144.02*	*3.93*
Cuenca (2008-2012)	149.44	N/A	N/A	N/A
Guayaquil (2008-2012)	144.02	N/A	N/A	N/A
South	*206.51*	N/A	N/A	N/A
Loja (2008-2010)	206.51	N/A	N/A	N/A
Pacific				
Manabi (2008-2012)	102.32	N/A	N/A	N/A
**France**	**258.36**	**290.55**	**174.68**	**44.85**
Metropolitan	*262.2*	*290.55*	*241.51*	*18.70*
Bas-Rhin (2008-2011)	259.58	N/A	N/A	N/A
Calvados(2008-2012)	262.18	N/A	N/A	N/A
Doubs (2008-2012)	249.02	N/A	N/A	N/A
Gironde (2008-2012)	271.17	N/A	N/A	N/A
Haut-Rhin (2008-2012)	253.26	N/A	N/A	N/A
Hérault (2008-2012)	260.05	N/A	N/A	N/A
Isère (2008-2012)	272.5	N/A	N/A	N/A
Lille-Métropole (2008-2012)	290.55	N/A	N/A	N/A
Limousin (2009-2012)	245.16	N/A	N/A	N/A
Loire-Atlantique (2008-2012)	263.22	N/A	N/A	N/A
Manche (2008-2011)	241.51	N/A	N/A	N/A
Somme (2008-2012)	258.65	N/A	N/A	N/A
Tarn (2008-2012)	247.29	N/A	N/A	N/A
Territoire de Belfort (2008-2012)	250.12	N/A	N/A	N/A
Vendée (2008-2012)	261.8	N/A	N/A	N/A
Overseas	*181.03*	*210.31*	*174.68*	*19.68*
Martinique (2008-2012)	174.68	N/A	N/A	N/A
French Guiana (2008-2012)	210.31	N/A	N/A	N/A
**Italy**	**264.22**	**308.74**	**221.44**	**33.04**
North-east	*281.59*	*308.74*	*238.48*	*25.33*
Ferrara (2008-2011)	297.89	N/A	N/A	N/A
Friuli Venezia Giulia (2008-2010)	270.77	N/A	N/A	N/A
Modena (2008-2012)	297.96	N/A	N/A	N/A

Parma (2008-2012)	308.74	N/A	N/A	N/A
Piacenza (2008-2011)	279.43	N/A	N/A	N/A
Reggio Emilia (2008-2012)	275.9	N/A	N/A	N/A
Romagna (2008-2012)	304.43	N/A	N/A	N/A
Sud Tirolo (2008-2010)	239.8	N/A	N/A	N/A
Trento (2008-2010)	238.48	N/A	N/A	N/A
Veneto (2008-2010)	268.13	N/A	N/A	N/A
North-west	*267.75*	*285.41*	*250.42*	*13.05*
Val d'Aosta (2008-2012)	250.42	N/A	N/A	N/A
Bergamo (2008-2012)	274.65	N/A	N/A	N/A
Biella (2008-2012)	285.41	N/A	N/A	N/A
Como (2008-2011)	266.94	N/A	N/A	N/A
Cremona (2008-2010)	270.61	N/A	N/A	N/A
Lecco (2008-2010)	278.7	N/A	N/A	N/A
Lombardy, South, Pavia (2008-2010)	262.47	N/A	N/A	N/A
Mantua (2008-2010)	285.36	N/A	N/A	N/A
Milan (2008-2012)	278.4	N/A	N/A	N/A
Monza (2008-2012)	264.69	N/A	N/A	N/A
Sondrio (2008-2012)	263.01	N/A	N/A	N/A
Turin (2008-2012)	254.76	N/A	N/A	N/A
Varese (2008-2012)	256.43	N/A	N/A	N/A
Central	*268.03*	*278.46*	*250.18*	*10.64*
Florence & Prato (2008-2010)	278.46	N/A	N/A	N/A
Latina (2008-2012)	250.18	N/A	N/A	N/A
Umbria (2008-2011)	269.74	N/A	N/A	N/A
South	*240.80*	*248.79*	*233.13*	*6.50*
Barletta (2008-2011)	240.82	N/A	N/A	N/A
Caserta (2008-2010)	233.13	N/A	N/A	N/A
Naples (2008-2012)	241.35	N/A	N/A	N/A
Taranto (2008-2011)	248.79	N/A	N/A	N/A
Islands	*237.76*	*247.23*	*221.44*	*10.94*
Catania, Messina & Enna (2008-2012)	247.1	N/A	N/A	N/A
Nuoro (2008-2012)	247.23	N/A	N/A	N/A
Palermo (2008-2012)	231.35	N/A	N/A	N/A
Ragusa & Caltanissetta (2008-2012)	221.44	N/A	N/A	N/A

Sassari (2008-2011)	246.68	N/A	N/A	N/A
Syracuse (2008-2012)	221.66	N/A	N/A	N/A
**Peru, Lima**	**187.51**	N/A	N/A	N/A
**Portugal, Azores**	**209.67**	N/A	N/A	N/A
**Spain**	**210.56**	**230.08**	**178.33**	**24.58**
North-East	*216.12*	*225.98*	*182.05*	*19.44*
Asturias (2008-2010)	210.62	N/A	N/A	N/A
Basque Country (2008-2012)	230.08	N/A	N/A	N/A
Castellón (2008-2012)	186.35	N/A	N/A	N/A
Girona (2008-2012)	208.84	N/A	N/A	N/A
La Rioja (2008-2012)	199.38	N/A	N/A	N/A
Mallorca (2008-2011)	202.42	N/A	N/A	N/A
Navarra (2008-2010)	229.29	N/A	N/A	N/A
Tarragona (2008-2012)	220.01	N/A	N/A	N/A
South	*200.68*	*230.08*	*178.33*	*25.54*
Albacete (2008-2010)	182.05	N/A	N/A	N/A
Canary Isles (2008-2011)	199.72	N/A	N/A	N/A
Ciudad Real (2008-2011)	197.91	N/A	N/A	N/A
Cuenca (2008-2011)	178.33	N/A	N/A	N/A
Granada (2008-2012)	201.59	N/A	N/A	N/A
Murcia (2008-2010)	214.03	N/A	N/A	N/A
**Switzerland**	**261.41**	**270.26**	**255.11**	**5.80**
Fribourg (2008-2012)	258.44	N/A	N/A	N/A
Geneva (2008-2012)	270.26	N/A	N/A	N/A
Neuchâtel (2008-2012)	256.49	N/A	N/A	N/A
Ticino (2008-2012)	262.59	N/A	N/A	N/A
Valais (2008-2012)	255.11	N/A	N/A	N/A
Vaud (2008-2012)	260.17	N/A	N/A	N/A
**Uruguay**	**216.77**	N/A	N/A	N/A
**Puerto Rico**	**197.95**	N/A	N/A	N/A

*Countries in bold, regions in italics, cancer registries in normal font. Regions may be a group of geographically related cancer registries or a single registry (if responsible for wide within region incidence variation).

** Registry responsible for /R ≥30 in country/region

N/A: geographic entity not split into sub-entities because r/R <30.
